# Cardiometabolic health, prescribed antipsychotics and health-related quality of life in people with schizophrenia-spectrum disorders: a cross-sectional study

**DOI:** 10.1186/s12888-016-1121-1

**Published:** 2016-11-18

**Authors:** Daniel Bressington, Jolene Mui, Mei Ling Tse, Richard Gray, Eric F. C. Cheung, Wai Tong Chien

**Affiliations:** 1 School of Nursing, The Hong Kong Polytechnic University, Kowloon, Hong Kong; 2 Castle Peak Hospital, Tuen Mun, Hong Kong; 3 Health Services Research Centre, Hamad Medical Corporation, Doha, Qatar

**Keywords:** Schizophrenia, Cardiovascular disease risk, Cardiometabolic health, Antipsychotics, Polypharmacy, Defined Daily Dose, QRISK®2, Obesity, Quality of life, SF-12

## Abstract

**Background:**

People with schizophrenia–spectrum disorders (SSD) often have high levels of obesity and poor cardiometabolic health. Certain types of antipsychotics have been shown to contribute towards weight gain and there is some equivocal evidence that obesity is related to poor health-related quality of life (HRQoL) in people with SSD. It is also still uncertain if antipsychotic polypharmacy/higher doses of antipsychotics are linked with HRQoL and/or increased risk of obesity/Cardiovascular Disease (CVD). Therefore, this study aimed to examine potential relationships between prescribed antipsychotic medication regimens, cardiometabolic health risks and HRQoL in community-based Chinese people with SSD.

**Method:**

This cross-sectional study reports the results of baseline measurements of a random sample of patients in an ongoing controlled trial of physical health intervention for people with severe mental illness. Data from these randomly-selected participants (*n* = 82) were analysed to calculate 10-year CVD relative-risk (using QRISK®2 score), estimate the prevalence of metabolic syndrome and contextualize patients’ prescribed antipsychotics (types, combinations and Daily Defined Dose equivalent). Patients self-reported their HRQoL (SF12v2) and their obesity condition was assessed by waist-circumference and Body Mass Index (BMI).

**Results:**

Two-thirds of patients had a BMI ≥23 kg/m^2^, almost half were centrally obese and 29% met the criteria for metabolic syndrome. The individual relative-risk of CVD ranged from 0.62 to 15, and 13% had a moderate-to-high 10-year CVD risk score. Regression models showed that lower physical HRQoL was predicted by higher BMI and lower mental HRQoL. Higher Defined Daily Dose, clozapine, younger age and male gender were found to explain 40% of the variance in CVD relative risk.

**Conclusion:**

The findings indicate that cardiometabolic health risks in people with SSD may be more common than those reported in the general Hong Kong population. The results also provide further support for the need to consider antipsychotic polypharmacy and higher doses of antipsychotics as factors that may contribute towards cardiometabolic risk in Chinese patients with SSD. Clinicians in Hong Kong should consider using routine CVD risk screening, and be aware that younger male patients who are taking clozapine and prescribed higher Defined Daily Dose seem to have the highest relative-risk of CVD.

**Trial registration:**

Clinicaltrials.gov NCT02453217. Prospectively registered on 19^th^ May 2015.

## Background

The poor cardiometabolic health state of people diagnosed with schizophrenia–spectrum disorders (SSD) and other severe mental illnesses (SMI) is a major clinical concern and a priority area for health services improvement worldwide [1–3]. It is now well recognised that compared with the general population people with SMI have a higher risk of developing obesity-related issues such as hypertension, stroke, metabolic syndrome (MES), and type-2 diabetes [4–8]. For example, it has been regularly reported that the risk of obesity in people with SMI is at least two times greater than in the general population [4].

In addition to obesity making a significant contribution towards the reduced life expectancy of people with SMI [9, 10], it may also negatively impact on patients’ quality of life; studies conducted in the United States and Canada have shown that lower levels of physical health-related quality of life (HRQoL) in people with SSD are related to higher Body Mass Index (BMI) and waist circumference measurement [11–13]. Although physical HRQoL has been shown by some studies to be linked with obesity-related measures in SSD, a similar connection between mental HRQoL and body weight is not consistently reported in the literature [12, 14]. The relationship between MES and reduced HRQoL in people with SSD has also been investigated in Europe and the United States, with some studies reporting a significant association [15], whilst others identified no such connection [16].

Cardiovascular diseases are also commonly seen, and poorly treated in people with SSD. A meta-analysis involving 13 studies demonstrated that schizophrenia is associated with increased incidence of total cardiovascular disease (CVD), stroke, coronary heart disease (CHD) and congestive heart failure, with pooled relative-risks ranging from 1.2 to 1.81 [8]. The survival rates of SSD patients with CHD are lower than the general population with CHD; Harris and Barraclough [17] reported 90% more deaths and Lahti et al. [18] reported a 2.9 relative risk of mortality when compared to people without a SMI who were diagnosed with CHD. Despite the increased prevalence of CVD, some studies have also shown that compared to the general population patients with SMI are less likely than to receive surgical interventions for CHD [19], have medications prescribed for CVD [20], and/or receive CVD risk screening tests [21].

The concerns about the increased prevalence of CVD in people with SSD have led to international calls for regular routine screening for CVD in this patient group [2, 22–25]. In line with these recommendations, a number of structured CVD risk assessment tools are now used within health screening programmes for people with SMI [1, 26]. The QRISK®2 [27, 28] and the Framingham coronary heart disease risk score [29] have both been used to measure CVD risk scores in this patient group in a variety of clinical settings [1, 30]. Very few such studies have been conducted in Asian countries; one example is Tay, Nurjono and Lee [31] who used the Framingham risk score to determine the 10 year CVD risk of patients with SSD in Singapore. The results of this study demonstrated that the SSD participants had a mean 10 year CVD risk of 4.6% compared to 3.1% in the non-SSD comparison group. The QRISK®2 tool is now increasingly used instead of the Framingham score [30] because it incorporates additional risk factors (for example, ethnicity, past history of smoking and family history of CVD). One UK study using the QRISK®2 found that patients with SMI in high-security inpatient forensic settings were more than twice-as-likely to have a high risk of CVD compared to people in the community without a SMI [28].

Numerous factors are likely to negatively influence the cardiometabolic health of people with SSD; including poor access to health services, unhealthy lifestyle behaviours, genetic predispositions and treatment-related issues [4, 32, 33]. Although the potential iatrogenic effects of treatment are only one factor that is likely to interact with a range of other influences on physical health, previous studies have consistently reported that antipsychotic medication is associated with increased risk for a range of cardiometabolic disorders [34, 35]. A large systematic review [36] also convincingly demonstrates that some antipsychotics are associated with at least twice the risk of MES, when compared with un-medicated people with SMI. Although individual antipsychotic medications are clearly associated (in varying degrees) with weight gain and type 2 diabetes [37], the relationships between the dosages, types and combined use (polypharmacy) of antipsychotic drugs and cardiometabolic health problems is not so clear. There is conflicting evidence from some cohort studies that the duration of exposure to antipsychotic medication, use of antipsychotic polypharmacy and higher antipsychotic dosages are linked to increased cardiovascular-related mortality in people with SSD [38–40]. A recent study conducted with Thai patients with SSD reported that BMI was significantly greater in patients prescribed antipsychotic polypharmacy as opposed to those prescribed monotherapy [41]. Nevertheless, it is still uncertain if a direct relationship exists between antipsychotic polypharmacy and weight gain in all patients with SSD [42, 43]; whereas systematic reviews have highlighted that antipsychotic polypharmacy is most likely to be associated with greater weight gain in younger people [5, 44].

There are very few studies conducted globally examining the potential relationships between cardiometabolic health risks, HRQoL and doses/combined use of prescribed antipsychotics. Therefore, the first objective of this study was to establish the level of CVD relative risk (using QRISK®2) and prevalence of obesity (BMI and waist circumference) in community-based Chinese people prescribed antipsychotic medications for SSD. The second objective was to examine potential relationships between prescribed antipsychotic treatment/clinical characteristics, CVD risk levels, and HRQoL in the study participants.

## Methods

### Design

This observational, cross-sectional descriptive study reports the results of the baseline measurements of a random sample of patients (*n* = 82) participating in a randomised controlled trial of physical health intervention for Chinese people with severe mental illness. The ongoing trial was prospectively registered online at Clinicaltrials.gov (reference number NCT02453217). We report the current study in accordance with the Strengthening the Reporting of Observational Studies in Epidemiology (STROBE) guidelines [45]. The RCT was designed to test the effectiveness and acceptability of the Chinese Health Improvement Profile (CHIP) intervention compared to treatment as usual on patients’ self-perceived physical well- being over a 12-month period. The RCT was necessary in order to robustly test the promising effects of the CHIP intervention observed in our earlier prospective case series study.

### Study setting

The study was conducted in a Community Psychiatric Service (CPS) in the New Territories, the largest geographical region of Hong Kong SAR, China. The multi-disciplinary community psychiatric team provided both crisis intervention and longer-term, recovery-focused community psychiatric services for people with different types of mental health problems.

### Ethical issues

The study was approved by the Clinical Research Ethics Committee of Hospital Authority, Hong Kong and the Human Subjects Research Ethics Committee of The Hong Kong Polytechnic University prior to the start of the study. Patients were required to give written informed consent to take part in the study and for permission to access their medical records. The participants’ information sheets clearly stated voluntary participation and rights of withdrawal from the study at any point without any negative treatment-related consequences.

### Recruitment and selection

Patients were recruited for the ongoing cluster randomized controlled trial (RCT). The participant inclusion criteria for the RCT were patients aged 18–65 years with an ICD10 diagnosis of a SMI (schizophrenia and other related psychotic disorders, bipolar disorder, or major depression). A total of 510 patients with SMI were screened from the caseloads of 12 Community Mental Health Nurses (CMHNs). Potential participants (*n* = 155) were excluded if they were judged as being incapable of providing informed consent, currently an inpatient, or if they were diagnosed with co-morbidity of another chronic medical/or mental disorders such as learning disability, substance misuse disorders and organic brain diseases. A list of 15 eligible patients from each of the CMHNs caseloads was randomly generated using an external online randomization service (sealedenvelope.com); and they were approached until a minimum of 140 had agreed to take part. This minimum sample size was calculated for the RCT (assuming a medium-large effect size for the primary outcome measure, a significance level of 0.05 and a potential attrition rate of 30%). A total of 23 patients (14% of 165 approached) did not agree to participate and one patient was recruited but later found to be ineligible, resulting in 141 patients recruited into the RCT. We did not record the demographic or clinical characteristics of those patients who refused to participate, consequently we are unable to ascertain if they differed significantly from patients who agreed to take part.

For the current nested observational study, we analysed data from outpatients with any types of SSD who were aged 25–65 years and prescribed antipsychotic medication. We wanted to focus on patients with SSD in order to directly compare the findings with other SSD studies, and as the rates of CVD risk and obesity vary greatly in accordance with the type of SMI diagnosis we did not include data from patients with a diagnosis of bipolar disorder, and/or major depression. It was also necessary to exclude patients <25 years old in order to improve the predictability of QRISK®2 scores (the minimum age that can be entered into the QRISK®2 calculator is 25 and therefore the algorithm would underestimate the relative risk of patients aged <25 years). Subsequently, 59 patients were excluded as ineligible due to age, diagnosis, or not being prescribed antipsychotics; resulting in a final sample of 82 participants.

### Data collection procedures and measures

The 12 CMHNs undertook data collection as baseline measurements for the cluster RCT either in the CPS clinic or at patients’ homes. All recruitment and baseline measurements were completed before the randomisation of the CMHNs into the intervention or treatment-as-usual group. Data were collected from July to September 2015.

#### Demographic and clinical information

The CHMNs recorded patients’ height, weight, BMI, waist circumference measurement, heart rate (radial pulse), and blood pressure. Blood pressure was recorded using a digital sphygmomanometer whilst participants were in a seating position, weight was measured using a digital scale and a digital stadiometer was used to measure height. The CMHNs were instructed to record waist circumference whilst patients were stood in a relaxed position using a measuring tape placed snugly across the midpoint between the lower rib and the top of the iliac crest at a level parallel to the floor. In addition, all recent (≤1 year) clinical data relating to the physical state of participants (i.e. temperature, respiration rate, urine tests, fasting blood glucose levels, lipid/cholesterol levels, liver function tests and serum prolactin levels) that were routinely recorded as part of standard practice in the electronic patient records system were also examined and recorded. Prevalence of obesity was calculated with gender/ethnicity specific waist circumference measurements and the BMI (kg/m^2^). Data relating to smoking status, presence of rheumatoid arthritis, diabetes, heart and kidney diseases, and family history of CVD were obtained from the patients or their medical records.

#### Prescribed antipsychotic treatment

Types of antipsychotics, dosage and formulations currently prescribed to patients were recorded by the CHMNs from the information in the medical records/prescription charts. To facilitate the direct comparison across patients on different antipsychotics and identify/examine the relationship between the dosages and the CVD risk/obesity, a standardized and reliable unit of dosage measurement was necessary (i.e. the Defined Daily Dose (DDD) [46]. The system of DDD is defined as “*the assumed average maintenance dose per day for a drug used for its main indication in adults*” and is recommended as an international standard for drug utilization studies by the World Health Organization [47]. A global average is assigned as the single DDD for each drug and route of administration. For long-acting injections, the DDDs are based on the average recommended doses divided by the dosing interval. Prescribed dosages of antipsychotics for patients was converted into multiples of the Defined Daily Dose (DDD) for each individual antipsychotic by dividing the prescribed daily dose by the DDD. For patients on more than one antipsychotic, the multiples of DDD for each drug were summed up to give a cumulative dosage measurement.

#### Cardiovascular risk scores

The version 2 of QRISK® (QRISK®2-2015) [27, 28] was used to calculate the 10-year CVD risk score of each participant. The QRISK®2 score and an associated individual CVD relative risk (relative to a person of the same age, sex and ethnicity without clinical indicators of risk and a cholesterol ratio of 4.0, systolic blood pressure of 125 and an ethnicity-specific healthy BMI) were estimated by entering a number of factors into the online QRISK®2 -2015 calculation tool (https://qrisk.org/2015/). These factors included: age, sex, ethnicity, height, weight, smoking status, systolic blood pressure, cholesterol/high-density lipoprotein (HDL) ratio, hypertension treatment, presence of rheumatoid arthritis, diabetes, heart and kidney diseases, and family history of CVD. The test results recorded closest in time to the anthropometric measures were used to calculate cardiovascular risk scores. Where test results were unavailable, or more than 1-year-old, the values were left blank and missing items were automatically imputed by the QRISK®2 calculator with predicted values based on the patient’s ethnicity, age and sex. The QRISK®2 algorithm was developed from the QResearch medical database of 2 million patients collected at 550 general practices in UK from 1993 to 2008 and is updated annually to reflect the actual changes of the population. Compared to Framingham score, the equation of QRISK®2 was derived from more recent and diverse ethnic groups (and the derivative cohort consisted of almost 20,000 people of Chinese ethnicity). It was also based on a larger variety of data (having additional data on ethnicity, family medical history, socio-economic status, and other clinical factors). The predictability of QRISK®2 was validated in samples of the QResearch database [27] and external data sources [48, 49] and compared with the corresponding Framingham scores. The results showed QRISK®2 outperformed Framingham score in respect to discrimination (QRISK®2 predicted 38% of the variation in men and 43% in women compared with Framingham’s 35 and 39% respectively). In 2010 the UK’s National Institute for Health and Care Excellence guidelines stated that the Framingham risk algorithm should not be used for CVD risk assessment unless it was considered together with other approaches (such as QRISK®2) [50], and the UK’s Royal College of Psychiatrists now suggest to use the QRISK®2 to estimate CVD risk in people with SSD [1].

#### Prevalence of metabolic syndrome (MES)

The consensus criteria of metabolic syndrome (MES) for Chinese populations from the International Diabetes Federation (IDF) [36] were used. Three out of five parameters, including central obesity (waist circumference of ≥80 cm for females and ≥90 cm for males) plus two additional ones from the following four aspects, including: raised blood pressure (systolic BP ≥130 or diastolic BP ≥85 mm/Hg), reduced HDL (<1.03 mmol/L in males, <1.29 mmol/L in females), raised triglycerides (≥1.7 mmol/L) and elevated fasting plasma glucose (≥ 5.6 mmol/L), were used to define the presence of MES in the participants.

#### Health-related quality of life

Patients self-reported their perceived HRQoL using the widely used and well-established 12-Item Short Form Survey 2nd version (SF12v2). Physical HRQoL was calculated using the physical component subscale (PCS) and mental HRQoL with the mental component subscale (MCS). The SF12v2 was developed by Ware, Kosinski, and Dewey [51] as a shorter version of the previous SF36 [52]. The Hong Kong Chinese version of the SF12v2 demonstrated good test–retest reliability and internal consistency (intraclass correlation 0.82; Cronbach’s alpha 0.67) for a Chinese adult population [53].

### Data analysis strategy

IBM® SPSS® software version 21 for Windows® was used for all analyses. The clinical characteristics, prescribed antipsychotic medication, prevalence of obesity and metabolic diseases, cardiometabolic risk levels, and HRQoL among the participants were summarised by descriptive statistics. Relationships between these above and the other health parameters were explored by correlation and tests of between group differences. Parametric (i.e. independent sample T (two-tailed) or ANOVA) and non-parametric (i.e. Mann-Whitney U or Kruskal-Wallis) tests were used to examine the differences in BMI/waist circumference, QRISK®2 score/CVD relative risk, and HRQoL scores across different antipsychotic medication usage groups in terms of their: number of types of antipsychotics (1-3), antipsychotic polypharmacy (yes/no,) antipsychotic combinations (first generation, second generation, first and second generation, long-acting intramuscular injection, oral, and both oral and long acting intramuscular injection), and Clozapine and DDD ranges. Multiple step-wise linear regression analyses were performed to identify which of the statistically significant treatment-related and clinical/patient-related variables could satisfactorily explain the variance in physical HRQoL and CVD relative risk. The level of significance was set at *p* < .05 for all tests.

## Results

### Demographic and clinical characteristics

The demographic and clinical characteristics of participants are summarised in Table 1.

**Table 1:** Demographic, clinical and treatment characteristics of study participants (*N* = 82)

Demographics		
Male – N (%)		49 (59.8)
Female		33 (40.2)
Age in years – Mean (SD), range		48.67 (8.57), 30–65
Duration of illness in months – Mean (SD), range		204.03 (138.12), 4–720
Numbers of previous psychiatric admissions –Mean (SD), range		3.97 (4.17), 0–20
Marital Status – N (%)		
Single		33 (40.2)
Married		28 (34.1)
Divorced		11 (13.4)
Other		10 (12.3)
Educational Level – N (%)		
Primary		26 (31.7)
Secondary		44 (53.7)
University/college		9 (11.0)
None/other		3 (3.6)
Employment Status – N (%)		
Full time		19 (23.2)
Part time		5 (6.1)
Unemployed		40 (48.8)
Other		18 (22.0)
Psychiatric Diagnosis – N (%)		
Schizophrenia		40 (48.8)
Paranoid schizophrenia		33 (40.2)
Other psychosis (including schizoaffective/delusional disorder, psychosis NOS)		9 (11.0)
Smoking status – N (%)		
Non smoker		50 (61.0)
Ex-smoker		1 (1.2)
Light smoker (<10 per day)		5 (6.1)
Moderate smoker (10–19 per day)		11 (13.4)
Heavy smoker (≥20 per day)		15 (18.3)
Medication Treatment – N (%)		
Prescribed an antihypertensive		25 (30.5)
Prescribed a statin		3 (3.7)
Prescribed diabetes treatment		7 (8.5)
Prescribed antipsychotic polypharmacy		28 (34.1)
Number of Antipsychotics Prescribed – N (%)		
1		54 (65.9)
2		24 (29.3)
3		4 (4.9)
Prescribed psychotropic medications – N (%)		
First Generation Antipsychotics		35 (42.7)
Second Generation Antipsychotics (all types)		60 (73.2)
Olanzapine		16 (19.5)
Risperidone		9 (11.0)
Paliperidone		9 (11.0)
Quetiapine		7 (8.5)
Amisulpride		4 (4.9)
Aripiprazole		2 (2.4)
Clozapine		14 (17.1)
Clozapine and another antipsychotic		5 (6.1)
First and Second Generation Antipsychotics		12 (14.6)
Long acting intramuscular antipsychotic injection		31 (37.8)
Oral antipsychotic and long acting intramuscular injection		19 (23.2)
Mood stabilizer/anti-manic drugs		
Lithium		3 (3.7)
Sodium valproate		7 (8.5)
SSRI antidepressant		2 (2.4)
Prescribed Dosage of Antipsychotics		
Cumulative multiples of DDD – Mean (SD), range		1.62 (1.08), 0.1–5.14
≤1.0 DDD – N (%)		32 (39.0)
1.1–2.0 DDD – N (%)		26 (31.7)
>2.0 DDD – N (%)		24 (29.3)
Cardiometabolic Health		
BMI (kg/m^2^) – Mean (SD)		24.81 (3.73)
BMI underweight – N (%)		3 (3.7)
BMI healthy – N (%)		22 (26.8)
BMI overweight – N (%)		18 (22.0)
BMI obese I – N (%)		33 (40.2)
BMI obese II – N (%)		5 (6.1)
Waist circumference obese (≥80 cm females, ≥90 cm males) – N (%)		40 (48.8)
Metabolic syndrome (meets IDF criteria) – N (%)		24 (29.3)
QRISK®2 10-year CVD risk score – Mean (SD)		5.28 (5.45)
<10% CVD risk score (low) – N (%)		71 (86.6)
10–19.9% CVD risk score (moderate) – N (%)		9 (11.0)
20% + CVD risk score (high) – N (%)		2 (2.4)
10-year CVD relative risk	– Mean (SD)	2.80 (2.96)
	– Median (IQR)	1.80 (1.87)
Health-Related Quality of Life		
Physical component score (PCS) – Mean (SD)		46.01 (7.11)
Mental component score (MCS) – Mean (SD)		46.93 (9.59)

*BMI* body mass index, *CVD* cardiovascular disease, *DDD* daily defined dose, *IDF* international diabetes federation, *SD* standard deviation, *NOS* not otherwise specified

Data from a total of 82 participants with a mean age of 49 years (SD = 8.57, range 30–65) were analysed. Nearly 90% of the patients were diagnosed with schizophrenia for more than 10 years and had an average of 4 hospital admissions. In spite of the relatively high prevalence of metabolic syndrome (29%), very few were receiving diabetes (8.5%) or cholesterol treatments (3.7%). Around one-third were on antihypertensive medication (*n* = 25), however only seven were diagnosed with hypertension, with the majority (*n* = 18) being prescribed propranolol for controlling anxiety or extrapyramidal side effects. A total of 28 patients (34%) were current smokers.

About one-third of the patients were prescribed more than one type of antipsychotic (antipsychotic polypharmacy). Most were on second-generation antipsychotics (SGA); however, about 15% were on both first-generation antipsychotics (FGA) and SGA. Over one-third were prescribed long-acting intramuscular injections (LAI), and around a quarter was taking both LAIs and oral formulations. According to the Defined Daily Dose (DDD) suggested by the World Health Organisation, 61% of patients were prescribed antipsychotics in excess of the recommended adult DDD (>1.0 cumulative DDD).

### Cardiometabolic health & health-related quality of life

The cardiometabolic health risks and HRQoL scores of participants are presented in Table 1. Cardiometabolic health risks were observed to be very common; over two-thirds were found to have an unhealthy BMI (≥23 kgm^2^), nearly half were obese in terms of both BMI and waist circumference, and 29% met the IDF criteria of metabolic syndrome. In terms of QRISK®2 scores, certain participants (13%) showed a moderate-to-high CVD risk score (≥10%); whereas, the overall 10-year CVD individual relative risk was high with a mean of 2.8 (SD = 2.96) and median of 1.8 (IQR = 1.87). Nevertheless, there was a wide range of 10 year CVD relative risk (between 0.62 and 15) among the patients.

### Relationships between demographic/clinical characteristics, cardiometabolic health risks and HRQoL

The correlations between demographic/clinical characteristics, cardiometabolic health and HRQoL are presented in Table 2. Factors significantly related to BMI included a weak negative correlation with age (*p* = .045) and weak positive correlation with number of previous admissions (*p* = .015). Age was also negatively correlated with CVD relative risk level (*p* < .001). Waist circumference measurement was positively correlated number of previous hospital admissions (*p* < .001). The two SF12v2 subscale scores were correlated to each other (*p* = .006) and increased BMI was negatively correlated with PCS (*p* = .032). No other significant correlations were found between demographic/clinical characteristics, cardiometabolic health risks and HRQoL.

**Table 2:** Demographic and clinical characteristics correlated with body mass index, waist circumference, relative risk of cardiovascular disease, HRQoL summary subscales and diastolic blood pressure

	BMI	Waist circumference	CVD relative risk	PCS	MCS	Diastolic blood pressure
Age in years (*n* = 80–82)	^a^-.220, .049*	^a^-.112, .315	-.386, < .001***	-.183, .099	.123, .269	.116, .299
Number of previous psychiatric admissions (*n* = 77–78)	.276, .015*	.386, .001***	.165, .149	-.182, .111	-.136, .236	.209, .067
Number of antipsychotic types (*n =* 81–82)	.330, .003**	.171, .124	.270, .014*	-.123, .270	-.138, .217	.180, .106
Dosage of antipsychotics (DDD) (*n =* 81–82)	.257, .021*	.214, .054	.361, .001**	-.149, .182	-.092, .413	.244, .027*
PCS (*n =* 81–82)	-.239, .032*	-.118, .291	-.005, .967	*-*	.299, .006**	-.047, .678
MCS (*n =* 81–82)	.093, .409	.118, .289	.030, .789	.299, .006**	*-*	.077, .492

*BMI* body mass index, *CVD* cardiovascular disease, *DDD* daily defined dose, *MCS* mental component score, *PCS* physical component score

Variables in the table are Spearman’s rho (*r*_*s*_) or ^a^Pearson’s r (*r*), followed by *p*-value

* *p* < .05, ** *p* < .01, *** *p* < .001

### Relationships between prescribed antipsychotics, HRQoL and cardiometabolic health risks

The main correlations between prescribed antipsychotics, cardiometabolic health and HRQoL are shown in Table 2. Greater numbers of types of prescribed antipsychotics were significantly positively correlated with increased BMI (*p* = .003) and elevated 10-year CVD relative-risk (*p* = .014). Cumulative DDD was positively correlated with BMI (*p* = .021), CVD relative risk (*p* = .001) and diastolic blood pressure (*p* = .027). There were no other significant correlations identified between prescribed antipsychotics, cardiometabolic health risks and HRQoL.

### Differences in HRQoL across demographic/clinical characteristic groups and antipsychotic treatment regimens

Females had a higher MCS scores than males (Z = -2.30, *p* = .022) with mean ranks of 36.54 (male) and 48.86 (female), but a significant gender difference was not observed in the PCS scores. No significant relationships were identified between HRQoL and any other demographic/clinical characteristic or antipsychotic treatment groups.

### Differences in cardiometabolic health across demographic/clinical characteristic groups

Table 3 shows the differences in cardiometabolic health and HRQoL across demographic and clinical characteristic groups. The presence of MES was only associated with higher number of previous admissions (mean ranks = 44.37 vs 32.81, *p* = .027) but not any other demographic factor and clinical characteristic. Males had a greater CVD relative risk than females (*p* < .001).

**Table 3:** Differences in cardiometabolic health risk indicators across gender and antipsychotic treatment groups

	BMI (kg/m^2^)				Waist circumference (cm)				Diastolic blood pressure			Pulse			CVD relative risk		
	MD^a^	[95% CI]	*T*	*p*	MD^a^	[95% CI]	*T*	*p*	MR^b^	*Z*	*p*	MR^b^	*Z*	*p*	MR^b^	*Z*	*p*
Gender	1.21	[0.46, 2.88]	1.44	.154	3.25	[-1.84, 8.34]	1.27	.208	46.24 (male)	−2.22	.027*	44.76 *(male)*	−1.15	.130	49.32 (*male*)	−3.62	< .001***
									34.45 (female)			36.67 *(female)*			29.89 (*female*)		
Clozapine prescribed *(yes Vs no)*	0.85	[-1.41, 3.11]	0.75	.456	3.07	[-3.60, 9.74]	0.92	.363	41.52 *(no)*	−0.02	.985	56.04 (yes)	−2.52	.012*	55.71 (*yes*)	−2.45	.014*
									41.39 *(yes)*			38.51 *(no)*			8.57 (*no*)		
Oral and LAI *(combination Vs single route)*	2.58	[0.71, 4.46]	2.74	.008**	4.30	[-1.60, 10.20]	1.45	.151	50.82 *(combine)*	−1.96	.050	42.46 *(single)*	−0.67	.505	53.68 (*combine*)	−2.54	.011*
									38.69 *(single)*			38.32 *(combined)*			37.83 (*single)*		
1^st^ and 2^nd^ generation antipsychotics *(combination Vs single class)*	3.01	[0.77, 5.25]	2.67	.009**	2.98	[-4.13, 10.9]	0.83	.407	49.54 *(combine)*	−1.28	.202	42.20 *(single)*	−0.65	.519	59.50 (*combine*)	−2.83	.005**
									40.12 *(single)*			37.42 *(combine)*			38.41 (*single)*		
	MD^c^	[95% CI]	*F*	*p*	MD^c^	[95% CI]	*F*	*p*	MR^d^	*H*	*p*	MR^d^	*H*	*p*	MR^d^	*H*	*p*
Number of antipsychotic types *(one, two, three)*	5.25	[0.73, 9.77]	4.93	.010*	5.43	[-9.13, 19.98]	0.47	1.000	*49.44 (two)*	*3.83*	*.147*	43.45 *(one)*	1.14	.564	37.19 *(one*)	6.71	.035*
	*(one vs three)*				*(one, two, three)*				*38.50 (three)*			38.23 *(two)*			47.52 *(two)*		
									*38.19 (one)*			34.75 *(three)*			63.50 *(three)*		
Dosage of prescribed antipsychotics *(>2 DDD, 1.1–2 DDD, ≤1 DDD)*	3.94	[1.57, 6.30]	8.73	.001***	9.73	[2.22, 17.24]	5.28	.007**	*49.90 (>2)*	*7.77*	.021*	*42.66 (*≤1)	0.13	*.940*	52.04 *(>2)*	9.41	.009**
	*(>2 vs 1.1–2)*				*(>2 vs 1.1–2)*				*44.44 (1.1–2)*			*40.81 (>2)*			42.90 *(1.1–2)*		
	2.79	[0.52, 5.06]	8.73	.011*					*32.81 (≤1)*			*40.71 (1.1–2)*			32.45 *(≤1)*		
	*(>2 vs ≤1)*																

*CI* confidence interval, *MD* mean difference, *MR* mean rank, *BMI* body mass index, *CVD* cardiovascular disease, *DDD* daily defined dose, *LAI* long acting intramuscular injection

^a^Independent sample T-test, ^b^Mann-Whitney *U*-test, ^c^1-way ANOVA (Bonferroni-corrected post-hoc test), ^d^Kruskal-Wallis test

* *p* < .05, ** *p* < .01, *** *p* < .001

### Differences in cardiometabolic health risks across antipsychotic treatment groups

Table 3 provides details of the test results for differences in cardiometabolic health between antipsychotic treatment groups.

#### BMI and central obesity

Patients prescribed a combination of oral and LAIs had a greater BMI than participants prescribed medications via a singular route of administration (*p* = .008). Patients prescribed a combination of both FGA and SGA also had a higher BMI compared to those on a single class of antipsychotic medication (*p* = .009). Bonferroni-corrected post-hoc tests of one-way ANOVA analysis revealed that patients prescribed with three different antipsychotic drugs had a significantly higher BMI than those prescribed with only one type, with a mean difference of 5.25 kg/m^2^ (*p* = .010).

A significant result of the ANOVA test revealed that patients prescribed with a dosage of >2.0 DDD had significantly higher BMI than those with dosages of 1.1–2.0 DDD or ≤1.0 DDD in the Bonferroni post-hoc test (*p* = .001 and *p* = .011 respectively). Higher multiples of DDD (>2.0 DDD) was also significantly associated with higher waist circumference compared to lower dosage (1.1–2.0 DDD) with a mean difference of 9.73 cm (*p*−.007) in Bonferroni post-hoc test.

#### Blood pressure and pulse

Kruskal-Wallis test showed significant differences of diastolic blood pressure across dosage groups, *p* = .021); Mann-Whitney U-tests indicate that difference between >2.0 DDD and ≤1.0 DDD groups was significant (*Z* = -2.61, *p* = .009, mean ranks of 35.02 vs 23.61). Patients prescribed clozapine had a higher diastolic blood pressure than those prescribed other antipsychotics (*p* = .012). Mann-Whitney U-tests of individual types of antipsychotics revealed a higher pulse rate for male patients than females (*p* = .027). No other significant differences were found in blood pressure and pulse across gender or antipsychotic treatment groups.

#### CVD relative risk

Patients prescribed a combination of oral antipsychotics and long acting intramuscular antipsychotic injections had a greater CVD relative-risk than those patients given antipsychotics via a singular route of administration (*p* = .011). Similarly, patients prescribed a combination of both FGA and SGA also had a higher CVD relative risk than those prescribed monotherapy (*p* = .005). Patients taking clozapine were found to have a greater CVD relative risk than those who were not prescribed clozapine (*p* = .014).

Kruskal-Wallis test of CVD relative risk across the antipsychotic dosage groups showed the differences were significant in patients prescribed with dosages over the WHO recommended DDD (*p* = .009). Mann-Whitney U-tests indicated that the risks of >2.0 DDD and ≤1.0 DDD groups were significantly different (Z = -3.20, *p* = .001) with mean ranks of 36.54 and 22.47 respectively. Significant differences in CVD relative risk across number of antipsychotic types were also found using Kruskal-Wallis test (*p* = .035); Mann-Whitney *U*-test showed that the CVD relative risk of one type of antipsychotic (mean rank = 28.2) is significantly less from that of three types (mean rank = 47.0, *Z* = -2.15, *p* = .032).

### Regression analysis of physical HRQoL and CVD relative-risk

Stepwise linear regression analysis was performed to assess the associations of BMI and MCS with PCS subscale (of HRQoL) scores. Collinearity test confirmed the independency of BMI and MCS (tolerance = .992, VIF = 1.008) and assumption of independent errors of residuals was met (Durbin-Watson value = 1.574). Standard residual plots showed that the data contained no outliers and met the criteria of normality. The model (*F* (2, 78) = 5.03, *p* = .009) with R^2^ of .114 and adjusted R^2^ of .091 showed that both BMI and MCS were significant predictors. Beta-coefficients and *p*-values are shown in Table 4.

**Table 4:** Results from multiple regression models: A) Physical health-related quality of life (HRQoL) regressed on body mass index and mental HRQoL. B) Relative risk of cardiovascular disease regressed on age, gender, clozapine medication and antipsychotic dosage

	*b*	SE	Beta	*p*	Adjusted *R* ^*2*^
A) PCS regressed on:					
BMI (kg/m^2^)	−0.516	0.203	−0.272	.013	
MCS	0.167	0.079	0.226	.038	
Constant	50.918	6.025		< .001	
					.091 (*p* = .009)
B) CVD relative risk regressed on:					
Prescribed DDD	0.198	0.063	0.283	.002	
On clozapine	0.523	0.175	0.263	.004	
Age (years)	−0.030	0.008	−0.346	< .001	
Gender (male = 1)	−0.364	0.138	−0.238	.010	
Constant	1.751	0.515		.001	
					.399 (*p* < .001)

*b* unstandardized regression coefficient, *Beta* standardized regression coefficient, *SE* standard error, *BMI* body mass index, *CVD* cardiovascular disease, *DDD* daily defined dose, *MCS* mental component score, *PCS* physical component score

Another stepwise linear regression was conducted to explore the significant predictors of CVD relative risk. The relative risk was studied instead of QRISK®2 score because it was age, sex and ethnicity controlled and this could provide more accurate results. Given the ratio nature of the CVD relative risk variable and non-normal distribution, we used transformed log-normal scores in the stepwise linear regression. Explanatory treatment variables that were found to be significantly associated with CVD relative risk (including multiples of DDD, number of antipsychotic types, combinations of oral and LAI, combinations of FGA and SGA, clozapine prescription) and possible demographic variables (age and gender) were entered into the model. Collinearity tests indicated that the VIF of these seven variables were all <5 and thus multi-collinearity was not a concern. A significant model including age, gender, DDD and clozapine were found to explain 39.9% of the variance in CVD relative risk (adjusted R^2^ = .399, *F* (4, 77) = 14.45, *p* < .001). Table 4 summarizes the details of beta-coefficients of each contributing variable. Analysis of residuals confirmed the data contained no outliers and assumption of independent errors (Durbin-Watson value = 1.921), normality, homogeneity of variance and linearity were all met.

## Discussion

The first objective of this study was to establish the prevalence of cardiometabolic risks in the study population (schizophrenia spectrum disorders; SSD) as indicated by levels of overweight/obesity, CVD relative risk and presence of MES. Over two-thirds of participants (68%) were found to have a BMI ≥23 kg/m^2^ and almost half were centrally obese. Recent estimates have suggested that 39% of the Hong Kong population has a BMI ≥23 kg/m^2^ [54] and thus our results indicate that the patients in the current study have a 174% increased risk of being overweight/obese when compared with the general population. An earlier study conducted by Guo et al., in 10 sites across mainland China [55] reported that 54% of 896 patients with schizophrenia had a BMI ≥23 kg/m^2^; compared to the 43% observed in the general Chinese population [56] this equates to an increased risk of 126%. Therefore, it is possible that people with SSD in Hong Kong face greater health inequalities compared to their mainland Chinese counterparts. However, there are numerous potential alternative reasons for these disparities in the results, such as all participants in Guo et al.’s study being prescribed only one type of antipsychotic, and a younger mean age and shorter duration of illness than participants in our current study.

The prevalence of metabolic syndrome (MES) in the current study is 29% as opposed to 17% of people in the general Hong Kong population [57, 58], suggesting a potential increased risk for MES of 170% for Chinese people with SSD in Hong Kong. The rate in the current study in randomly selected patients with schizophrenia is lower than the 35% observed in an earlier Hong Kong study of a convenience sample of people with SMI [59]. The differences between these rates of MES may relate to selection bias in the earlier study [59], in which the non-randomised (convenience) sample were invited to participate by their clinicians on a sequential basis. The variations in rates of MES, levels of obesity and CVD risks across different populations of people with SSD are now well-documented; for example, prevalence rates range between 19% and 68% dependent on their country, ethnicity, age, gender, prescribed medications, MES criteria used and duration of illness [4, 60]. Despite these differences, the rates of MES are consistently observed to be elevated in people with SSD when compared with the general population [60].

In the current study over 13% of patients were found to have a moderate-to-high 10-year CVD risk score (as indicated by >10% on the QRISK®2), which is considerably higher than the 8% reported in the large UK derivation cohort of people without SMI but less than the 16% observed in a community-based UK people with SMI [28]. Similarly, based on a QRISK®2 mean score of 5.28% in our study and the mean Framingham scores of 8.5% in American patients with schizophrenia [35] (in spite of different calculation methods for the QRISK®2 and Framingham scores), our results suggest that people with SSDs in Hong Kong have a reduced CVD risk compared to the US patients. Perhaps these findings are not surprising given that the CVD rates and life expectancies of the general populations of Hong Kong, the UK and the US vary considerably. Although there are great differences in average life expectancies, it is consistently reported that SSD patients have mortality rates 2–2.5 higher than the general population across international settings, and this has led to calls from the World Health Organisation (WHO) to improve the prevention, identification, assessment and treatment of physical health in people with SSD across the globe [25].

The second objective was to examine potential relationships between prescribed treatment/clinical characteristics, CVD relative risk and health related quality of life (HRQoL). Patients’ self-perceived physical HRQoL (PCS) and mental HRQoL (MCS) were both lower than the reported population norms of 50.2 (PCS) and 50.1 (MCS) in Chinese adults in Hong Kong as measured by the SF-12v2 [61]. Therefore, the HRQoL of participants in this study suggests mild-moderate levels of perceived physical and mental health concerns. In line with previous studies we found a relationship between increased BMI and lower physical HRQoL [12, 62]. In fact, BMI was the strongest predictor of physical HRQoL in the regression model (beta coefficient of -.272, *p* = .013), providing further evidence that reductions in BMI may result in improvements in physical HRQoL. However, no direct relationship was observed between mental HRQoL and BMI, which is consistent with results from other studies in people with SMI [12, 14]. It is also interesting to note that although mental HRQoL was not found to directly relate to measures of body weight, the regression model revealed that MCS was a significant explanatory variable for the variance in physical HRQoL subscale scores (beta coefficient of .226, *p* = .038). Therefore, the findings highlight a potential significant relationship between perceived mental and physical health, and indicate that weight reduction interventions in this patient group could have the potential to not only enhance physical HRQoL, but also to indirectly improve mental HRQoL of these patients.

Unlike some previous studies [15] which found that reduced HRQoL was associated with metabolic syndrome (MES), we did not find any differences in HRQoL between the patients who did and who did not meet the criteria for MES. Nor did we find any differences in self-reported HRQoL in patients with low/high CVD relative-risk, or identify significant correlations between HRQoL and CVD relative risk. This is an important finding because it may indicate that despite having an elevated risk of CVD or MES, patients do not perceive their physical HRQoL as being low, and thus they may be less likely to actively seek treatment and/or be motivated to make lifestyle changes to improve their physical health state. This reinforces the need to actively promote routine cardiometabolic health assessment for all patients with SSD and share concerns about identified risks using effective psychoeducational interventions. We also did not find any relationship between HRQoL and antipsychotic Defined Daily Dose (DDD); which is corresponded with a recent European study of patients with schizophrenia of a similar mean duration of illness [13]. This also could suggest that patients who are receiving higher dosage(s) of antipsychotic do not subjectively recognise any negative impacts on their physical or mental HRQoL, as such they may be less likely to complain about being on higher doses and thus might require appropriate screening and support to recognise potential problems. These observations, in conjunction with evidence suggesting that people with severe mental illness are less likely to receive CVD risk screening tests and are undertreated for CVD [19–21] highlight that mental health care providers need to be particularly vigilant in monitoring the cardiometabolic health of people with schizophrenia, and should proactively promote treatment as appropriate.

A large proportion of patients were prescribed antipsychotic polypharmacy (34%) and cumulative doses of antipsychotics that are in excess of the WHO DDD (61%). This is surprising given that with the exception of clozapine augmentation [63–65] there is a lack of convincing evidence that antipsychotic polypharmacy is any more effective than monotherapy in improving psychotic symptoms. This is also concerning because our results show that antipsychotic polypharmacy and a higher DDD was associated with increased CVD relative risk, a greater BMI, higher waist circumference measurement and elevated diastolic blood pressure. These results support the findings from some earlier studies which reported that metabolic changes seen in people with SSD are greater in those prescribed polypharmacy [43] and are often antipsychotic dose-dependent [66]. The 34% of patients prescribed antipsychotic polypharmacy in our study is similar to the median percentage of 32% for Asian countries reported in a large systematic review which pooled data from 147 studies in four geographical regions [67]. The same review also reports the median percentages of antipsychotic polypharmacy use in Europe (23%), the US (16%) and Oceania (16.4%). Therefore, our study results seem to be consistent with earlier studies suggesting that staff in Asian settings may be more likely to prescribe antipsychotic polypharmacy than their counterparts in other areas of the world. This may highlight a need to review local clinical/prescribing guidelines, critically review patients’ prescriptions more frequently or consider staff training requirements. However, this is a cross-sectional study and thus it is impossible to ascertain the appropriateness of the use of more than one antipsychotic drug for participants; it is possible that some polypharmacy prescriptions were made for justifiable reasons, such as the cross-titration of antipsychotic medications, the use of co-prescribed aripiprazole to reduce weight-gain or the augmentation of clozapine to improve symptom response [68].

The regression model in this study also revealed that DDD, clozapine, age and male gender explained almost 40% of the variance in participants’ CVD relative risk. Age was found to be the strongest predictor of CVD relative risk in the regression model (coefficient of -.346, *p* < .001), which could possibly be explained by previous observations that BMI makes a direct contribution towards CVD risk and that antipsychotic-induced weight gain is most marked in younger people [5, 44]. These findings also replicate results from some earlier studies, which indicated that antipsychotics appeared to directly contribute towards CVD risk [69, 70] and also that higher doses/polypharmacy are associated with an increased risk of cardiometabolic disorders [38]. In the current study it is difficult to be certain which combinations of antipsychotics present the highest cardiometabolic risks because the duration of exposure to different antipsychotic treatment regimens was not recorded. However, the results certainly seem to highlight that psychiatrists in Hong Kong should aim to proactively minimise the dosages and reduce the number of different antipsychotics that are prescribed for people with SSD.

## Study limitations

This study has a number of methodological limitations which could negatively impact on the validity and generalisability of the findings. Although the participants were randomly selected from the CMHNs caseloads the narrow inclusion criteria used to select the subgroup of patients analysed in the current cross-sectional study may have introduced some selection bias. The 10 year CVD risk estimates should be treated with some caution; despite the QRISK®2-2015 calculation tool having an option for Chinese ethnicity, the measure was not validated for Chinese people with SSD living in Hong Kong. We were also unable to objectively verify that the CMHNs adhered to the suggested approach towards waist circumference measurement and missing (or outdated) blood test data were automatically imputed based on age, sex and ethnicity-specific predicted values. Other possible influences on the relationships between antipsychotics and cardiometabolic health (such as levels of medication adherence, duration of exposure to antipsychotic regimens and lifestyle behaviours) are potential unmeasured confounding variables. In addition, the relatively small sample size may have increased the risk of errors and the cross-sectional design is unable to demonstrate causal relationships. Future research should therefore adopt a prospective cohort study design which aims to examine the potential relationships between antipsychotic treatment, obesity, HRQoL and CVD risk in a larger sample of randomly selected people with SSD.

## Conclusions

The 10-year CVD relative-risk estimates and high prevalence of obesity/metabolic syndrome suggest that Hong Kong Chinese people with SSD may experience worse cardiometabolic health than the general population. A lower physical HRQoL was associated with a higher BMI, and increased mental HRQoL was found to be related to greater physical HRQoL. Consequently, interventions that reduce obesity in this patient group could directly improve self-perceived physical health quality of life and indirectly enhance mental health quality of life. Clinicians in Hong Kong should consider using routine CVD risk screening for all patients with SSD, implement physical health promotion interventions as appropriate, and be aware that younger male patients who are prescribed higher doses of antipsychotics seem to have the most elevated relative risk of CVD. The relationships observed between cardiometabolic health and polypharmacy/higher doses provide further evidence that antipsychotic treatment regimens are modifiable factors that can contribute towards cardiometabolic risk and hence the subsequent patient mortality rates.

## Abbreviations

ANOVA: Analysis of variance
BMI: Body mass index
BP: Blood pressure
CHIP: Chinese health improvement profile
CMHN: Community mental health nurses
CPS: Community psychiatric service
CVD: Cardiovascular disease
DDD: Defined daily dose
HDL: High-density lipoprotein
HRQoL: Health-related quality of life
ICD10: International classification of diseases version 10
IDF: International Diabetes Federation
LAI: Long-acting intramuscular injections
MCS: Mental component subscale
MES: Metabolic syndrome
PCS: Physical component subscale
RCT: Randomized controlled trial
SF12: 12-item short form survey
SMI: Severe mental illness
SSD: Schizophrenia–spectrum disorders
